# A Prolonged Neurological Presentation of Legionnaire's Disease

**DOI:** 10.7759/cureus.15672

**Published:** 2021-06-15

**Authors:** Shaorinkumar Patel, Radhika Sharma, Barrett O Attarha, Pramod Reddy

**Affiliations:** 1 Internal Medicine, University of Florida College of Medicine – Jacksonville, Jacksonville, USA

**Keywords:** dysarthria, neurological symptoms, legionella, cerebellar symptoms, legionnaire's disease

## Abstract

Legionnaire’s disease is an uncommon pneumonic disease that can carry a mortality rate up to 20%. It commonly presents as an atypical lower respiratory illness. However, it is important to be mindful of the various extra-pulmonic presentations of the infection. Here, we present a case of a 51-year-old female who presented to the emergency department with lethargy, slurred speech, and confusion. Legionella has been reported to present with neurological symptoms but it is not a common occurrence in each hospitalization. These neurological symptoms often lead to an extensive workup and the complexity of the diagnostic workup can significantly influence patient outcome. However, it is crucial that physicians follow a systemic approach to establish a diagnosis in an expedited manner. This case emphasizes the importance of key objective evidence of legionella that can help guide a physician’s diagnostic approach.

## Introduction

Over the past two decades, the incidence of Legionnaire’s disease has increased over five-fold, with most cases presenting in the summer and early autumn [1]. It commonly presents as an atypical lower respiratory illness, however can lead to multi-organ involvement with a mortality of 60-70% without antibiotic therapy and 10-20% with therapy [2]. It can present with a wide-range of symptoms involving the renal, hepatic, gastrointestinal, and neurologic systems [3]. Legionella can manifest with neurological symptoms, however having an isolated neurological presentation is rare. Neurological symptoms consist of encephalopathy, cerebellar dysfunction, and focal deficits. Encephalopathy varies from mild confusion to coma which normally resolves with the resolution of the infection. However, there have been reports of persistent symptoms [4]. It is a difficult diagnosis to make due to its ambiguous clinical presentation without specific characteristics that suggest Legionella [5]. We report a case of a patient who presented with primarily neurological complaints of confusion, slurred speech, and an inability to follow commands, later diagnosed with Legionnaires’ disease.

## Case presentation

**Figure 1:**
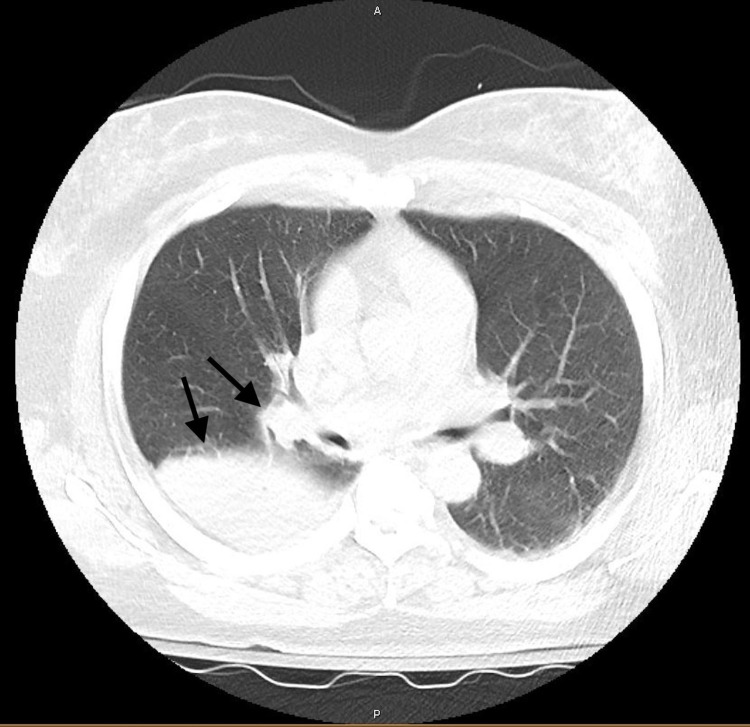
CTA Chest Right Upper Lobe CTA: Computed tomography angiography

A 51-year-old female with history of hypertension and human immunodeficiency virus (CD4 count 865, compliant on anti-retroviral therapy) presented to the emergency department for altered mental status. On initial examination, the patient was awake but confused, unable to follow commands, with dysarthria and noted to have diarrhea. Upon evaluation neurological exam was limited due to patient's confusion, however, the patient was moving all extremities, withdrawing pain stimuli, negative Babinski bilaterally, negative for facial droop, and intact pupillary reflex bilaterally. Workup was significant for right upper lobe consolidation seen on chest X-ray, leukocytosis with bandemia, transaminitis, elevated creatinine, hyponatremia, hypokalemia, and a significantly elevated creatine kinase level (Table 1). The patient was started on antibiotics for empiric coverage for meningitis and respiratory illness. However, differential also included stroke and infectious etiology. Further imaging was ordered which included a CT head and MRI brain which were both unremarkable for any mass effect or acute infarct and did not warrant a neurology consult at that time. The patient was later admitted to the intensive care unit after found to be hypotensive and hypoxic. In the intensive care unit (ICU), the patient was started on broad coverage with vancomycin, cefepime, and azithromycin for worsening hypoxia and severe sepsis. Patient's hypotension was fluid responsive and did not require any vasopressors to sustain her mean arterial pressure. Workup performed in the ICU included a negative lumbar puncture, computed tomography angiography of the chest that showed an obstructive mass in the right upper lobe (Figure 1), negative bronchoalveolar lavage, respiratory viral panel, and sputum culture. The pathogens tested included pneumocystis, tuberculosis, and fungal species. Cerebrospinal fluid was sent for herpes simplex virus testing and a culture of the fluid was performed which were both negative. Patient history of compliance on anti-retroviral therapy with a high CD4 count made human immunodeficiency virus associated infections less likely. Ultimately, the urine antigen was positive for Legionella.

**Table 1:** Labs Values on Day of Admission CBC: Complete Blood Count; CMP: Complete Metabolic Panel

CBC		CMP	
White Blood Cells (WBC)	12.63	Sodium	131
Red Blood Cells (RBC)	3.46	Potassium	2.3
Hemoglobin	10.9	Chloride	99
Hematocrit	31	Carbon dioxide total	18
Mean Corpuscular Value	89.3	Urea Nitrogen	20
Mean Corpuscular Hemoglobin	31.4	Creatinine	3.31
Mean Corpuscular Hemoglobin Concentration	35.2	Calcium	9.5
Red cell Distribution Width	14.7	Anion Gap	16
Platelet Count	151	Albumin	3.5
Neutrophils	64	Phosphorus	1.5
Bands %	24.3	Aspartate Aminotransferase (AST)	227
Sedimentation Rate	123	Alanine Aminotransferase (ALT)	120
Creatinine Kinase	15,007	Total Bilirubin	2.1
		Alkaline Phosphatase	309
CEREBROSPINAL STUDY			
RBC count	141		
WBC count	6		
Protein	37		
Herpes simplex virus PCR	Negative		
Fluid color	Colorless		
Gram Stain	No neutrophils Squamous epithelial cells organisms		

The patient's antibiotics were changed to Levofloxacin once all other infectious causes were ruled out. During the patient's stay in the ICU, she required short-term dialysis due to worsening rhabdomyolysis and oliguric acute kidney injury. An electroencephalogram was also performed due to minimal improvement of cognitive status and further showed encephalopathy of nonspecific etiology. With appropriate antibiotic therapy and improvement in renal function with short-term dialysis, the patient's confusion resolved and she was later extubated to room air. On discharge, she continued to have persistent dysarthria, oropharyngeal dysphagia, balance and coordination deficits. This was contributed to encephalopathy secondary to acute infectious process and metabolic changes with mild improvement noted during the stay with resolution of confusion. The oropharyngeal dysphagia could be explained by the prolonged intubation. Physical therapy recommended short-term rehabilitation. Family and the patient at that time agreed to outpatient physical therapy and speech therapy. Per primary care follow-up, the patient's dysarthria was persistent for two months post discharge with gradual improvement.

## Discussion

Legionella is unique due to its ability to present with a variety of different clinical presentations which can include significant neurological findings. These findings can consist of cerebral symptoms including confusion and cerebellar symptoms including gait, limb ataxia, and dysarthria [3]. The onset of neurological symptoms is roughly five days post pneumonic symptoms [6]. Though neurological symptoms are uncommon but not rare, the unique finding of cerebellar symptoms such as dysarthria in our patient is extremely rare and noted in limited case reports with a group study showing only 3.7% incidence rate [6]. Of these 3.7% of the cases, limb ataxia and speech disturbance were the leading symptoms. It is hypothesized that these manifestations of Legionnaire’s disease are a result of either direct bacterial tissue invasion or the toxic effect of released inflammatory factors [7].

Early diagnosis of Legionella has shown to decrease mortality [8]. The diagnosis of Legionella can be made with urine antigens, sputum cultures, and polymerase chain reaction. Urine antigens are a quick diagnostic tool with a sensitivity of more than 85% and specificity of more than 99% [8]. However, urine antigens only test the L. pneumophila serogroup. This is why it is vital to obtain sputum cultures and polymerase chain reaction testing which are both able to identify other serotypes. Once the diagnosis is made, the preferred treatment includes levofloxacin or azithromycin due to their bactericidal action, high intracellular concentrations, penetration of lung tissue, and activity against all Legionella serotypes. In our patient, the predominant presenting symptoms were neurologic and although it is rare for patients to have persistent and prolonged effects, our patient had persistent dysarthria for two to three months after discharge. This highlights the importance of an early diagnosis and prompt intervention to prevent prolonged complications.

## Conclusions

As reported, the incidence of cerebellar symptoms of Legionella is extremely rare but important to be mindful of. With the varying presentations of Legionella, neurological presentation is by far one of the most concerning symptoms on initial presentation due to its diagnostic complexity. These symptoms can be worrisome to clinicians leading to a broad differential and expensive workup. Increased awareness of this and other extrapulmonary manifestations of Legionella can allow for an efficient diagnosis and treatment of the disease process.
